# Phase Separation of Br-Doped CsPbI_3_: A Combined Cluster Expansion, Monte Carlo, and DFT Study

**DOI:** 10.3390/molecules29010092

**Published:** 2023-12-22

**Authors:** Prettier Maleka, Ratshilumela Dima, David Tshwane, Odireleng Ntwaeaborwa, Rapela Maphanga

**Affiliations:** 1 Next Generation Enterprises and Institutions, Council for Scientific and Industrial Research, P.O. Box 395, Pretoria 0001, South Africa; sdima@csir.co.za (R.D.); dtswane@csir.co.za (D.T.); rmaphanga@csir.co.za (R.M.); 2 School of Physics, University of the Witwatersrand, Private Bag X3, P.O. Box Wits 2050, Johannesburg 2050, South Africa; martin.ntwaeaborwa@wits.ac.za; 3 National Institute for Theoretical and Computational Sciences, Johannesburg 2000, South Africa

**Keywords:** cluster expansion, density functional theory, electronic properties, solar cells, inorganic perovskites, optical properties, thermodynamic properties

## Abstract

Cluster expansion, which is a method that describes the concentration-dependent thermodynamic properties of materials while maintaining density functional theory accuracy, was used to predict new (CsPbI_x_Br_1−x_) structures. The cluster-expansion method generated 42 new stable (CsPb)_x_I_y_Br_z_ (where x = 1 to 3 and y and z = 1 to 8) structures and these were ranked as meta-stable structures based on their enthalpies of formation. Monte Carlo calculations showed that CsPbI_0.5_Br_0.5_ composition separates into different phases at 300 K, but changes to a homogeneous phase at 700 K, suggesting that a different phase of CsPbI_3_ may exist at higher temperatures. Among the 42 predicted structures, randomly selected structures around iodide-rich, 50:50, and bromine-rich sites were studied further by determining their electronic, optical, mechanical, and thermodynamic properties using first-principle density functional theory. The materials possess similar properties as cubic Br-doped CsPbI_3_ perovskites. The mechanical properties of these compounds revealed that they are ductile in nature and mechanically stable. This work suggests that the introduction of impurities into CsPbI_3_ perovskite materials, as well as compositional engineering, can alter the electronic and optical properties, making them potential candidates for solar cell applications.

## 1. Introduction

The depletion of fossil fuels and the rapid growth in world population are the main drivers of research interest to find alternative renewable energy sources. Perovskite solar cells (PSCs) have been largely explored as a prospective source of clean and renewable energy. They have shown remarkable progress, with rapid increases in power conversion efficiency from early reports of approximately 3% in 2009 to over 25% today [1]. Despite their excellent optoelectronic properties, including tunable bandgap, high absorption coefficients, high carrier mobility, long diffusion lengths for electrons and holes, small effective masses, and a simple reproducible process, PSC devices must retain high efficiencies, while exhibiting decent stability and acceptable degradation for practical applications. Research on material-constituent mixing often concentrates on examining the stability of various phases, such as ordered intermediate ground states and end members, as well as investigating the miscibility gaps among them [2,3,4,5]. Certain intermediate phases can remain stable even at high temperatures and may prove advantageous for specific applications. For some purposes, disordered solid solutions, particularly mixed halides achieved through element mixing, are preferred, as they offer properties that can be adjusted easily. 

The perovskite structure tends to show enhanced stability in relation to phase transitions. The impact of halides on halide perovskites and their optical band gap is widely recognized. These halides provide an effective means of adjusting the band gap of these perovskites. For instance, the energy band gap can be changed from 1.5–2.0 eV by varying the types of halide ions present in the material [6,7,8]. By controlling the energy band gap, the absorption edge of the perovskite may be adjusted, which allows optimization of the efficiency and performance of the device. The X-site anions in perovskite materials directly affect the band edge states and determine the opto-electronic properties of the materials [9]. The most common X anions used in perovskites are halide anions F, Cl, Br, and I. These halides have eight valence electrons, and their respective ionic radii are 2.20, 1.96, 1.81, and 1.33 A for I, Br, Cl, and F, respectively. The decrease in ionic radius of the halide anion leads to a reduction in the bond length between the Pb atom and the X anion in a perovskite material. Consequently, the coupling of atomic orbitals between the Pb and the anion becomes stronger, resulting in increased overlap of the wavefunctions. This enhanced overlap has the effect of enlarging the energy band gap. In other words, the energy band gap of perovskite compounds becomes larger as the halide anion’s ionic radius decreases. For example, CsPbI_3_ has a reported band gap of approximately 1.73 eV [10], and that of CsPbBr_3_ is approximately 2.30 eV [11]. CsPbBr_3_ is regarded as less suitable for solar cell applications because of its large band gap. However, the thermal and phase stability of CsPbBr_3_ is better than that of CsPbI_3,_ which generated significant research interest. CsPbBr_3_ has been utilized as an active absorber in various perovskites solar cell (PSC) configurations and has power-conversion efficiency (PCE) up to 9% [9]. 

Most recently, significant attention has been focused on CsPbX_3_, where X represents I and Br, and they are often combined in various proportions to form CsPbI_3−x_Br_x_ (0 < x < 3). By adjusting the halogen ratio, compounds such as CsPbIBr_2_, CsPbI_2_Br, or non-integer ratios are formed. Various mixtures of Cs-based perovskites containing bromide and iodide, with improved thermal stability and reduced band gap, have been tested in perovskite solar cells [12,13]. This resulted in a notable increase in PCE, reaching approximately 16%. Beal et al. conducted the first photoluminescence (PL) experimental study on the light stability of mixed halide perovskite CsPbI_3−x_Br_x_ with a range of halide compositions [14]. The PL spectra of the films were discovered to be stable for 0 ≤ x ≤ 1 and demonstrated spectral redshift for 1 ≤ x ≤ 3 during the illumination procedure (intensity of 100 mW cm^2^). Chen et al. used fluorescence lifetime imaging to evaluate and obtain a better understanding of ion transport in CsPbI_2_Br films. The study was able to spot Br vacancies moving into dark regions, which led to the existence of I-phase photoluminescence elsewhere [15]. Phase segregation has the potential, under certain conditions, to have a significant impact on the performance of mixed halide perovskite photovoltaic devices. A thermodynamic model developed by Brivio et al. showed that MAPb(I_1−x_Br_x_)_3_ ranging from x = 0.3 and 0.6 can function as an extra energy source with light illumination at 300 K to help get beyond the kinetic energy barrier of halide segregation [16]. Lin et al. analyzed the electronic properties of CsPbI_3−x_Br_x_ to better understand the band gap structure of the mixed halogen perovskite crystal [17]. The calculated band gap of CsPbI_3−x_Br_x_ at atmospheric pressure showed that the crystal valence band maximum (VBM) and the conduction band minimum (CBM) are located at the Z-point, with high symmetry. This results in direct band-gap characteristics for various halogen ratios. The electron band gap demonstrated an increase and a blue-shift absorption edge when Br was incorporated, resulting in the varied electronegativities of the elements found in the CsPbI_3−x_Br_x_ perovskite materials. The presence of I-I and Pb-I bonding results in a Coulombic attraction. When the I^-^ is substituted with the Br^−^, there is transfer of electrons occurring between the two atoms because of their different electronegativities, causing a change in the total Coulomb energy and the resulting electron-transport characteristics.

Saliba et al. reported that replacing methylammonium with Cs^+^ and halides mixing can improve the electronic coupling across quantum dots, which, in turn, increases carrier mobility [18]. The stability of perovskites has been shown to improve with chemical intervention at grain boundaries; this may cause the ingress of water into the perovskite layer to be more difficult. The mixed-halide CsPbI_2_Br showed an enhanced Goldschmidt tolerance factor (0.84), as well as a better stability of the cubic phase, which paves the way for an incredibly stable all-inorganic PSC. The substitution of bromide in CsPbI_3_ reduces the temperature required for the formation of the CsPbI_2_Br black phase from 623 K to 523 K, and the CsPbI_2_Br exhibits improved stability compared to that of CsPbI_3_. By introducing Br ions into CsPbI_3_ perovskite using mixed halide composition via a two-step approach, Lee et al. reported experimental results with greatly improved ambient thermodynamic stability, exceeding that of hybrid perovskite [19]. 

The perovskite configuration offers outstanding tunability of the crystal, the electronic structure, the dimensionality, and the chemical content. Previous studies reported unconventional A_x_B_y_X_3z_ perovskites with impressive optoelectronic properties and stability in ambient conditions. Studies suggested that the perovskite formula can be altered into A_2_B_2_X_6_, A_3_B_3_X_9_, and A_3_B_2_X_9_ [20,21,22]. Saparov et al. characterized a unique lead-free Cs_3_Sb_2_I_9_ perovskite derivative as a potential candidate for high-band gap photovoltaics with an optical band gap of 2.05 eV [22]. Furthermore, attempts have been made to develop unique lead-free perovskites such as Cs_3_Bi_2_I_3_ [23], Cs_3_Cu_2_I_5_ [24], and Cs_4_SnBr_6_ [25]. Dong et al. reported on the geometrical, electronic, and optical properties of double-perovskite material Cs_2_NaMX_6_ (M = In, Tl, Sb, Bi; X = Cl, Br, I) using the first-principles calculations to investigate the halogen anions and metal anions affecting the properties [26]. The structural, opto-electronic, and mechanical properties of all-inorganic vacancy-ordered double-perovskite A_2_Sn_1−x_Ti_x_Y_6_ (A = K, Rb, Cs; Y = Cl, Br, I) were investigated using DFT, as reported by Chen et al. [27]. The band structure and optical absorption results demonstrated that all A_2_Sn_1−x_Ti_x_Br_6_ (A = K, Rb, Cs) materials are semiconductors with band gaps between 0.9–1.6 eV, and A_2_TiBr_6_ (A = K, Rb, Cs) compounds have the highest optical absorption both in the visible and ultraviolet regions, making them excellent materials for photovoltaic applications. Urmi et al. computed the opto-electronic properties of Cs_2_TiI_6_ (Cs_2_TiI_x_Br_6-x_) halide perovskite and the findings showed that this material has a considerable potential as a low-band gap alternative material for photovoltaic applications [28]. In this work, the cluster expansion (CE) method based on a genetic algorithm was used to predict new structures of Br-doped CsPbI_3_ and to further predict their structural, electronic, optical, mechanical, and thermodynamic properties using the first-principle calculations. As far as we are aware, theoretical investigation of the three structures with chemical compositions Cs_2_Pb_2_I_3_Br_3_, Cs_2_Pb_2_I_5_Br, and Cs_3_Pb_3_IBr_8_ have not been reported before. 

## 2. Results

### 2.1. Ground State Search

**Figure 1:**
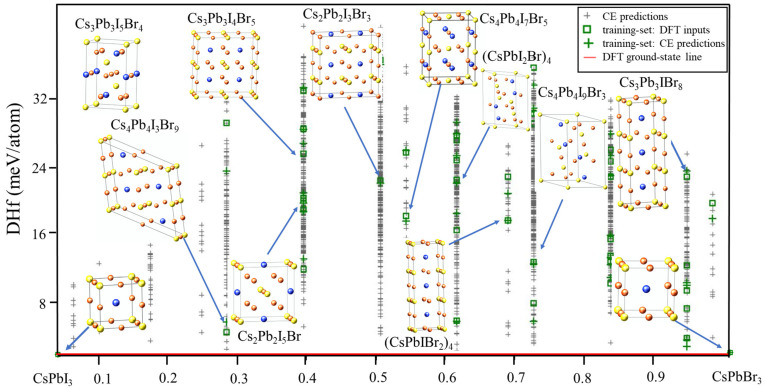
Ground-state diagram of CsPb(IBr)_3_.

To begin the search for the ground states of the CsPb(I/Br)_3_ system using CE, the DFT enthalpies of formation of CsPb(I/Br)_3_ were initially calculated. A total of 42 new structures suggested by the genetic algorithm (GA) were predicted. Using these structures, the GA was started and a 2 meV cross-validation score per atom was obtained. In the first run of CE, a new input structure was selected by the CE, which predicted it as a ground state. The total energy of the new structure was then calculated using VASP and added to the input set. Cluster-expansion predictions were modified and fitted in the procedure. The system was considered to have converged when no new ground-state structures were discovered. Figure 1 depicts the final ground-state line related to the FCC ground states of atoms and the predicted enthalpies of formation.

### 2.2. Miscibility Gap

From Table 1, it is apparent that there would be variations within the calculations because of the stochastic method used in the cluster-expansion method. However, based on the final line of the table (the 4th iteration), it can be inferred that the final CE includes 42 structures from the training set, with a CSV score of 0.652 meV/pos for the system. The next column remained unpopulated until the 2nd iteration, implying that the optimized scheme transitioned from the miscible constituents to the miscibility gap mode. Therefore, the system with a miscibility gap has no stable structures with respect to existing pure phases.

### 2.3. Monte Carlo

**Figure 2:**
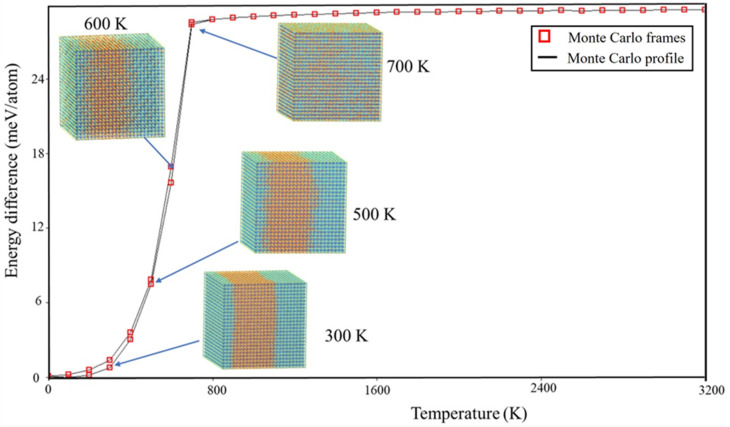
Temperature profile cross section through 25 × 25 × 25 Monte Carlo simulation for CsPb(IBr)_3_. Blue and orange balls are I and Br atoms, respectively.

**Figure 3:**
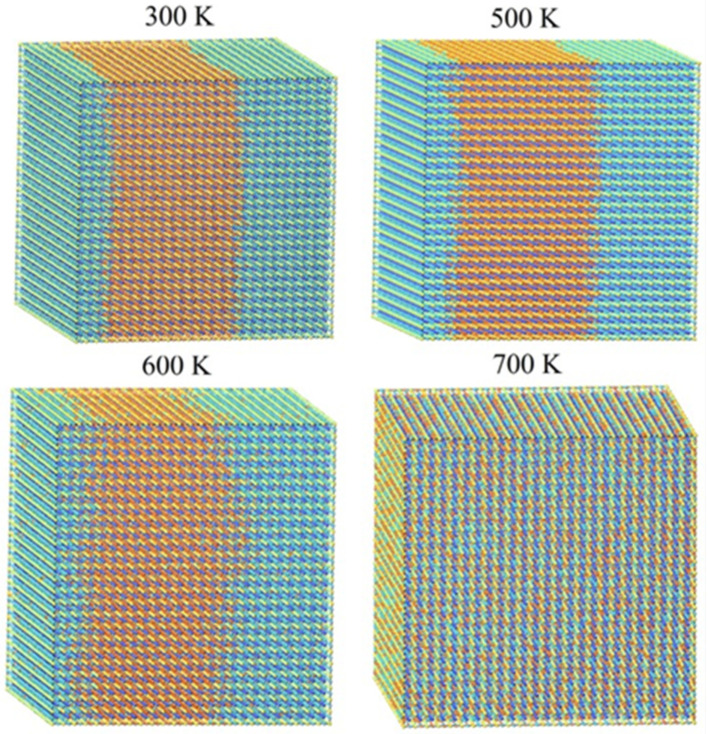
Monte Carlo simulation cells of CsPbI_0.5_Br_0.5_ at different temperatures. Blue and orange balls are I and Br atoms, respectively.

To visually examine the behavior of phase segregation in CsPbI_1−x_Br_x_ compounds, simulation cells with a fixed halides content (50.0% concentration) underwent annealing at temperatures that varied from 0 K to 3200 K. This annealing process was carried out using the canonical ensemble simulation technique called Monte Carlo. Observations were made of the spatial distributions of halides (I-Br) atoms within annealing simulation units. Figure 2 presents the spatial distribution patterns of I-Br atoms at four representative temperatures (300 K, 500 K, 600 K, and 700 K) for CsPbI_1−x_Br_x_ compounds, as these temperatures exhibited similar spatial morphologies. To enhance the visibility of the spatial distribution morphology, the figures include the display of Cs and Pb atoms in CsPbI_1−x_Br_x_ compounds. From the observations depicted in Figure 3, it is evident that there is a segregation of I and Br halide atoms in the considered CsPbI_1−x_Br_x_ compounds between 300 K and 500 K. This indicates that phase segregation can occur in CsPbI_1−x_Br_x_ compounds, leading to the separation of CsPbI_3_ (or I-rich CsPbI_1−x_Br_x_) and CsPbBr_3_ (or Br-rich CsPbI_1−x_Br_x_) at a low temperature. As the temperature increases, the agglomerated clusters gradually disperse at 600 K and the spatial distribution of the halides (I and Br) atoms becomes homogenous at 700 K. The homogeneous spatial distribution at elevated temperatures could be attributed to the increased thermal energy allowing the constituent atoms and ions (cesium, lead, and halides) to move more freely, filling the available space more uniformly.

### 2.4. Structural Properties

**Figure 4:**
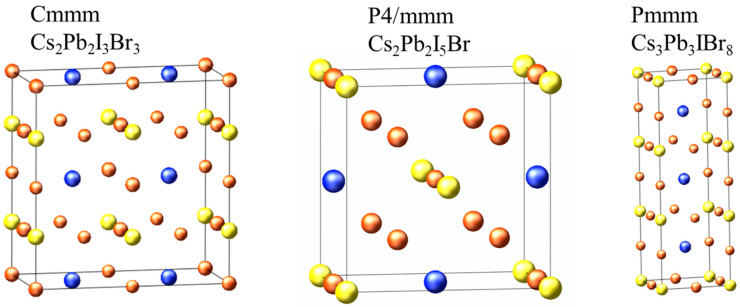
The cluster-expansion predicted structure of Cs_2_Pb_2_I_3_Br_3_, Cs_2_Pb_2_I_5_Br, and Cs_3_Pb_3_IBr_8_. Blue, yellow and orange balls are Cs, Pb and halides (I and Br) atoms, respectively.

From the 42 structures predicted by cluster expansion, we randomly selected three structures to investigate their structural, electronic, optical, elastic, and thermodynamic properties to predict their opto-electronic behavior. The random selection was mainly guided by previous studies on unconventional perovskites, indicating that the perovskite formula can be altered into A_2_B_2_X_6_, A_3_B_3_X_9_, and A_3_B_2_X_9_ phases, which we reported to be stable. The calculated structural parameters of the selected structures are shown in Figure 4 and Table 2. The lattice constants for Cs_2_Pb_2_I_3_Br_3_ and Cs_3_Pb_3_IBr_8_ are of an orthorhombic system, where a ≠ b ≠ c, whereas Cs_2_Pb_2_I_5_Br has tetragonal system lattice constants with a = b ≠ c, as shown in Table 2. The Pb-I bond length is longer than the Pb-Br bond length for Cs_2_Pb_2_I_3_Br_3_, Cs_2_Pb_2_I_5_Br, and Cs_3_Pb_3_IBr_8_ structures because of the difference in the ionic radii of Br and I. We could not find published experimental or theoretical data to compare our calculated structural properties for these predicted structures.

### 2.5. Electronic Properties

#### 2.5.1. Band Structure

**Figure 5:**
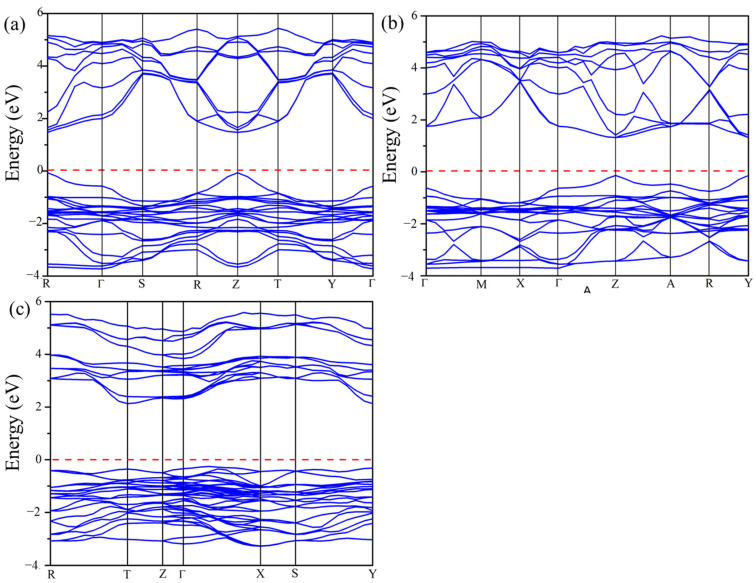
The calculated band structures of the predicted (**a**) Cs_2_Pb_2_I_3_Br_3_, (**b**) Cs_2_Pb_2_I_5_Br, and (**c**) Cs_3_Pb_3_IBr_8_ structures.

The calculated electronic band structures for Cs_2_Pb_2_I_3_Br_3_, Cs_2_Pb_2_I_5_Br, and Cs_3_Pb_3_IBr_8_ using the GGA-PBE functional are shown in Figure 5. The Fermi level represented with a horizontal red line was set at 0 eV to coincide with the VBM and CBM. For Cs_2_Pb_2_I_3_Br_3_ and Cs_2_Pb_2_I_5_Br, the VBM and CBM were observed at Z-point and at R point for Cs_3_Pb_3_IBr_8_, indicating direct band gap materials, as illustrated in Figure 5. The calculated band gaps of Cs_2_Pb_2_I_3_Br_3_, Cs_2_Pb_2_I_5_Br, and Cs_3_Pb_3_IBr_8_ were 1.692, 1.520, and 1.901 eV, as seen in Table 3. Again, there are no reported experimental or theoretical values to compare with. However, comparing these structures with the experimental band gap of CsPbI_3_ (1.73 eV) and CsPbBr_3_ (2.30 eV) demonstrated that mixing halogens can reduce the band gap of perovskites materials, making them suitable for solar cell applications [4,10]. 

#### 2.5.2. Density of States

**Figure 6:**
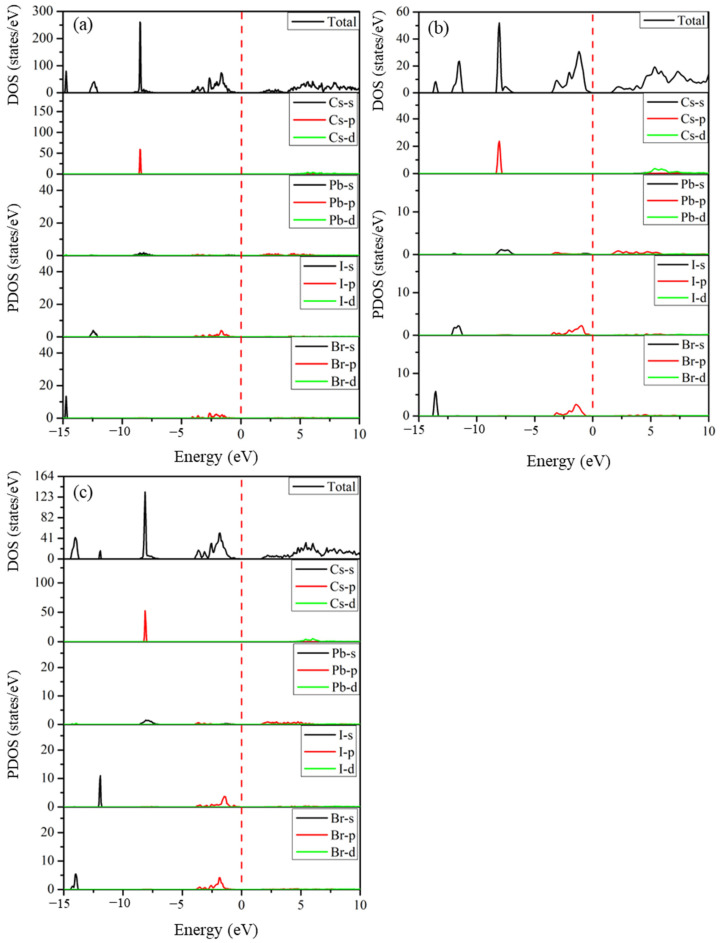
The calculated total and partial densities of states of the predicted (**a**) Cs_2_Pb_2_I_3_Br_3_, (**b**) Cs_2_Pb_2_I_5_Br, and (**c**) Cs_3_Pb_3_IBr_8_ structures.

The total and partial densities of states of the predicted structures Cs_2_Pb_2_I_3_Br_3_, Cs_2_Pb_2_I_5_Br, and Cs_3_Pb_3_IBr_8_, which determine the orbital contribution of atoms to the electronic states in the valence and conduction bands, are shown in Figure 6. The Fermi level represented with a vertical red line was set at 0 eV to coincide with the VB and CB. The valence band between −15.0 eV and −5 eV is made up of halides (I-5s and Br-4s orbitals) and a small contribution from Cs-5p and Pb-(4s and 4p) orbitals, as depicted in Figure 6. The valence upper band close to the Fermi level located from −5.0 eV to 0 eV shows contributions of Pb- (4s and 4p) and halides (I-5p and Br-4p) states. The conduction band is mainly comprised of Pb-4p orbitals near the band edge with a small contribution from the halide atoms (I-5p and Br-4p). These halogen atoms contribute to both valence and conduction bands, and this positively contributes to the electronic properties of the perovskite materials. These predicted structures have densities of states that are similar to those of CsPbI_3_ and CsPbBr_3._

### 2.6. Optical Properties

#### 2.6.1. Dielectric Function

Optical characteristics are necessary to determine the effectiveness of the photovoltaic properties of solar cell devices. These computed optical parameters are very important for perovskite materials because they determine the type of response these materials will show when the photons or light are incident upon them. The optical properties of the cubic perovskites CsPbI_3_, CsPbI_2_Br, CsPbBr_2_I, and CsPbBr_3_ were calculated using the frequency-dependent dielectric function, as follows:

(1) $$\varepsilon \left(\omega \right)=\varepsilon_{1}(\omega )+i\varepsilon_{2}\left(\omega \right)$$

where ε_1_(ω) and ε_2_(ω) are real and imaginary parts, respectively, of the dielectric function, ε(ω). Using the dielectric function ($\varepsilon_{1}\left(\omega \right)$ and $\varepsilon_{2}(\omega )$), other optical parameters, such as absorption coefficient α(ω), reflectivity R(ω), conductivity σ(ω), extinction coefficient K(ω), and refractive index n(ω) [29], can be determined using the following expressions:

(2) $$\alpha \left(\omega \right)=\sqrt{2\omega }{\left(\sqrt{\varepsilon_{1}^{2}(\omega )+\varepsilon_{2}^{2}(\omega )}-\varepsilon_{1}(\omega )\right)}^{\frac{1}{2}}$$

(3) $$n\left(\omega \right)=\frac{1}{\sqrt{2}}{\left(\sqrt{\varepsilon_{1}^{2}(\omega )+\varepsilon_{2}^{2}(\omega )}+\varepsilon_{1}(\omega )\right)}^{\frac{1}{2}}$$

(4) $$K\left(\omega \right)=\frac{1}{\sqrt{2}}{\left(\sqrt{\varepsilon_{1}^{2}(\omega )+\varepsilon_{2}^{2}(\omega )}-\varepsilon_{1}(\omega )\right)}^{\frac{1}{2}}$$

(5) $$R\left(\omega \right)={\left|\frac{\sqrt{\varepsilon \left(\omega \right)-1}}{\sqrt{\varepsilon \left(\omega \right)+1}}\right|}^{2}$$

(6) $$\sigma \left(\omega \right)=-\frac{i\omega }{4\pi }\varepsilon (\omega )$$

**Figure 7:**
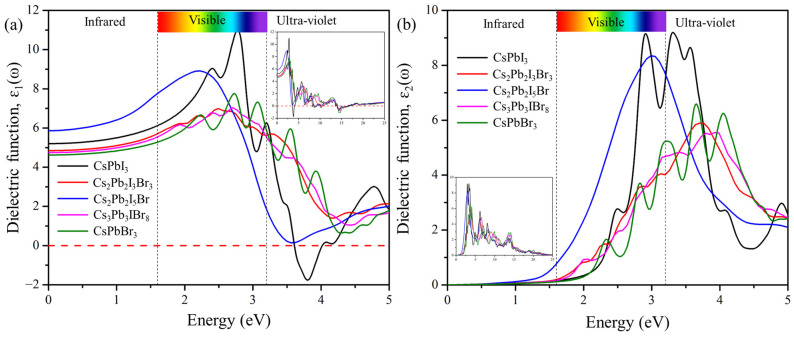
The calculated (**a**) real and (**b**) imaginary parts of pure phases (CsPbI_3_ and CsPbBr_3_) and the predicted structures (Cs_2_Pb_2_I_3_Br_3_, Cs_2_Pb_2_I_5_Br, and Cs_3_Pb_3_IBr_8_).

Figure 7 displays the real and imaginary parts of the dielectric function plotted against the frequency of the predicted structures Cs_2_Pb_2_I_3_Br_3_, Cs_2_Pb_2_I_5_Br, and Cs_3_Pb_3_IBr_8_. The static value of the ε_1_(0) defines the amount of carrier recombination and performance of the opto-electronic device [20]. The static values of the dielectric constant ε_1_(0) are 4.82, 5.88, and 4.80 for Cs_2_Pb_2_I_3_Br_3_, Cs_2_Pb_2_I_5_Br, and Cs_3_Pb_3_IBr_8_, respectively. These compounds display the most intense peaks at low energy (visible) regions. The calculated real part maximum values were 6.95 at 2.41 eV, 8.88 at 2.26 eV, and 4.41 at 2.96 eV for Cs_2_Pb_2_I_3_Br_3_, Cs_2_Pb_2_I_5_Br, and Cs_3_Pb_3_IBr_8_ respectively. The real-part dielectric function of these compounds has a higher peak in the visible region (low energy) but decreases in the ultra-violet region, which demonstrates that they are suitable for solar cell applications. It was also observed that these predicted structures are red shifted, compared to CsPbI_3_ and CsPbBr_3_.

The imaginary dielectric function in Figure 7b, ε_2_, begins at approximately 1.63 eV, 1.47 eV, and 1.82 eV for Cs_2_Pb_2_I_3_Br_3_, Cs_2_Pb_2_I_5_Br, and Cs_3_Pb_3_IBr_8_, respectively, which is related to the band gap of these compounds. As photon energy increases, the calculated imaginary part also increases, and reaches its highest peaks of 5.87 at 3.70 eV, 8.29 at 3.03 eV, and 5.53 at 3.86 eV for Cs_2_Pb_2_I_3_Br_3_, Cs_2_Pb_2_I_5_Br, and Cs_3_Pb_3_IBr_8_, respectively. Once these maximum values are reached, the ε_2_ of dielectric functions begins to drop, due to an increase in the energy of the photon. Cs_2_Pb_2_I_5_Br is the second most-intense peak, compared to others, indicating that mixed halides can also improve the intensity of the imaginary dielectric function.

#### 2.6.2. Refractive Index and Extinction Coefficient

**Figure 8:**
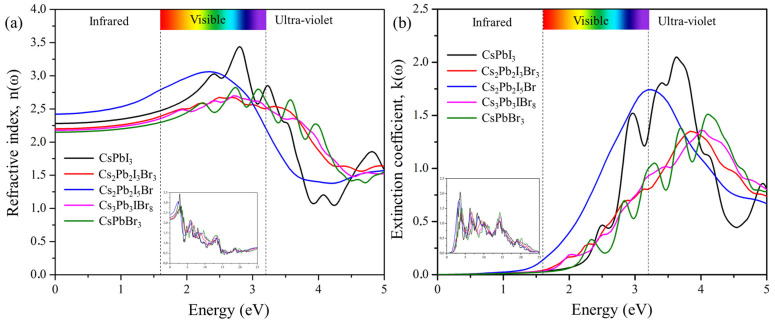
The calculated (**a**) refractive index and (**b**) extinction coefficient of the pure phase structures (CsPbI_3_ and CsPbBr_3_) and the predicted structures (Cs_2_Pb_2_I_3_Br_3_, Cs_2_Pb_2_I_5_Br, and Cs_3_Pb_3_IBr_8_).

The refractive index is defined by the amount of light bent or refracted when entering a material. Figure 8a shows the calculated refractive index as a function of photon energy for the predicted cluster-expansion structures Cs_2_Pb_2_I_3_Br_3_, Cs_2_Pb_2_I_5_Br, and Cs_3_Pb_3_IBr_8_. The static refractive indices n(0) are 2.20 for Cs_2_Pb_2_I_3_Br_3_, 2.43 for Cs_2_Pb_2_I_5_Br, and 2.19 for Cs_3_Pb_3_IBr_8_. The refractive indices increase and reach the maximum values of 2.67 at 2.51 eV for Cs_2_Pb_2_I_3_Br_3_, 3.06 at 2.36 eV for Cs_2_Pb_2_I_5_Br, and 2.69 at 2.72 eV for Cs_3_Pb_3_IBr_8_. The degree of light refraction is determined by the refractive index, which is an essential parameter that is particularly useful in photoelectric applications. The refractive index n(ω) is greater than 1 for all the compounds because photons interact with electrons, slowing down when entering a substance. The higher the refractive index, the greater the amount of slowing down that occurs when photons travel through the material. Typically, any mechanism that increases the electron density in a substance will also increase its refractive index. Cs_2_Pb_2_I_5_Br has the most intense peak, of around 2 eV, when compared to those CsPbI_3_ and CsPbBr_3_, indicating that mixed halides can also improve the intensity of the refractive index.

The ability of a material to reduce the amount of light that passes through it is referred to as the extinction coefficient, K(ω). Figure 8b demonstrates the calculated extinction coefficient for the predicted structures. The imaginary part of the refractive index, (n(ω)), is the extinction coefficient (K(ω)) of the materials. It is indicative of the radiation absorbed and has similar behavior an imaginary part, (ε_2_(ω)), of the dielectric function. The extinction coefficients, K(max), of the highest peaks were 1.37 at 6.71 eV for Cs_2_Pb_2_I_3_Br_3_, 1.73 at 3.24 eV for Cs_2_Pb_2_I_5_Br, and 1.34 at 4.12 eV for Cs_3_Pb_3_IBr_3_. Among the predicted structures, Cs_2_Pb_2_I_5_Br had the most intense peak in the visible region, compared to those of CsPbI_3_ and CsPbBr_3_.

#### 2.6.3. Reflectivity and Conductivity

**Figure 9:**
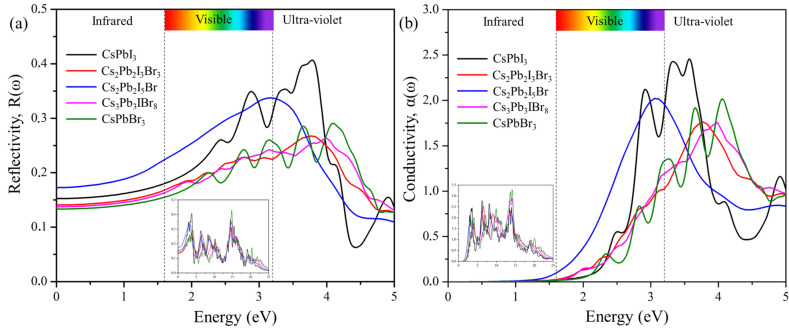
The calculated (**a**) reflectivity and (**b**) conductivity of the pure phase structures (CsPbI_3_ and CsPbBr_3_) and the predicted structures (Cs_2_Pb_2_I_3_Br_3_, Cs_2_Pb_2_I_5_Br, and Cs_3_Pb_3_IBr_8_).

For a photon of light incident on a material, we can determine how much light is reflected using reflectivity spectra, as shown in Figure 9a, which displays the plots of R(ω) against frequencies from 0 eV to 25 eV. The values of static reflectivity R(0) were observed to be 0.141 for Cs_2_Pb_2_I_3_Br_3_, 0.174 for Cs_2_Pb_2_I_5_Br, and 0.137 for Cs_2_Pb_2_I_5_Br. As the photon energy increases, the reflectivity also increases and reaches maximum, R(max), values of 0.308 at 14.699 eV for (Cs_2_Pb_2_I_3_Br_3_)_3_, 0.339 at 3.191 eV for Cs_2_Pb_2_I_5_Br, and 0.353 at 14.80 eV for Cs_3_Pb_3_IBr_8_. The optical conductivities, showing optically activated free charge carriers of the predicted structures as a function of incident photon energy, are depicted in Figure 9b. The maximum σ(ω) was observed at 6.55 eV for Cs_2_Pb_2_I_3_Br_3_, 13.51 eV for Cs_2_Pb_2_I_5_Br, and 14.184 eV for Cs_3_Pb_3_IBr_8_, and then it started to decrease with increasing photon energy. CsPbI_3_ had the most intense peak. Cs_2_Pb_2_I_5_Br had the second-most intense peak.

#### 2.6.4. Absorption Coefficient

**Figure 10:**
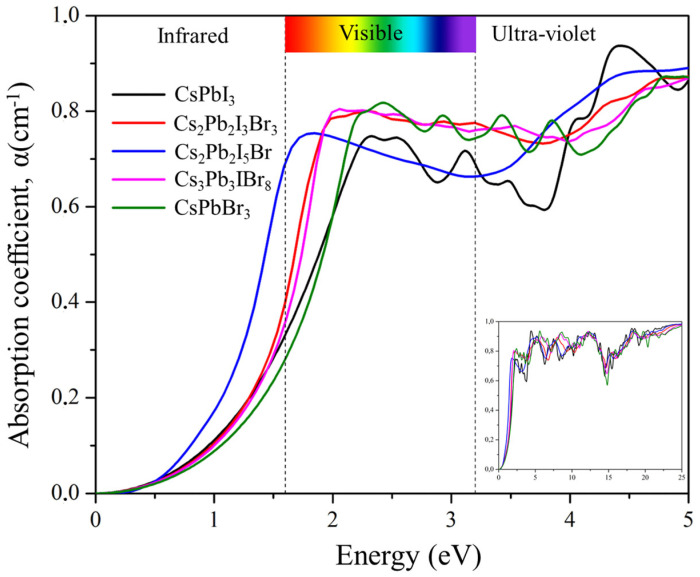
The calculated absorption coefficient of the pure phase structures (CsPbI_3_ and CsPbBr_3_) and the predicted structures (Cs_2_Pb_2_I_3_Br_3_, Cs_2_Pb_2_I_5_Br, and Cs_3_Pb_3_IBr_8_).

Figure 10 depicts the absorption coefficient α(ω) spectra of Cs_2_Pb_2_I_3_Br_3_, Cs_2_Pb_2_I_5_Br, and Cs_3_Pb_3_IBr_8_ as a function of photon energy. The absorption spectra demonstrate the penetration capability of photons passing through a material before absorption. The absorption spectra start below 2 eV due to the direct energy band gap of the materials. The strongest peaks were observed in the ultraviolet region at 18.33, 18.28, and 12.16 eV for Cs_2_Pb_2_I_3_Br_3_, Cs_2_PbI_5_Br, and Cs_3_Pb_3_IBr_8_, respectively. The primary absorption peak was attributed to charge-transfer excitation from the VB to the CB. Peak transitions in these compounds started from electrons of the empty orbital valence band, Cs 6 s and Pb 6p, to I 5p and Br 4p that dominated the deep conduction band. Mixing halogens (I and Br) depicted peaks that were similar to those of the two parent compounds (CsPbI_3_ and CsPbBr_3_). However, the predicted structures red-shifted optical absorption in the visible region suggested that these materials can be applied to solar cells.

### 2.7. Elastic Properties

The Cs_2_Pb_2_I_5_Br material belongs to the tetragonal system and has six independent elastic constants, C_11_, C_12_, C_13_, C_33_, C_44_, and C_66_, while Cs_2_Pb_2_I_3_Br_3_ and Cs_3_Pb_3_IBr_8_ belong to orthorhombic system with nine elastic constants, C_11_, C_12_, C_13_, C_22_, C_23_, C_33_, C_44_, C_55_, and C_66_, as shown in Table 3. These elastic constants determined the mechanical stability of compounds. Using Equations (7) and (8), the elastic constants of these structures were determined, and they suggested that these predicted structures were mechanically stable. The corresponding mechanical stability criterion for tetragonal crystal structure is defined as:

(7) $$C_{44}>0,C_{66}>0,C_{11}>\left|C_{12}\right|\;\mathrm{and}\;C_{11}+C_{12}-\frac{2C_{13}^{2}}{C_{33}}>0$$

The corresponding mechanical stability criterion for orthorhombic crystal structure is defined as:

(8) $$C_{ii}>O;i=1-6\;C_{ii}+C_{jj}-{2C}_{ij}>0;i,j=1,2,3\;C_{11}+C_{22}+C_{33}+2(C_{12}+C_{13}+C_{23})>0$$

The bulk modulus and shear moduli are crucial characteristics of crystals that demonstrate their capability to resist compression and shear deformations. Table 4 presents the elastic constants, bulk modulus, shear modulus, Young’s modulus, Pugh’s ratio, and Poisson’s ratio of the predicted structures Cs_2_Pb_2_I_3_Br_3_, Cs_2_Pb_2_I_5_Br, and Cs_3_Pb_3_IBr_8_. It was evident that Cs_2_Pb_2_I_3_Br_3_ has a high bulk, shear, and Young’s moduli. This indicated that the Cs_2_Pb_2_I_3_Br_3_ compound had relatively good resistance to deformation, a good capability to resist shear strain, and good rigidity. All the B/G and Poisson ratios for these materials were higher than 1.75 and 0.26, indicating that the materials are ductile [19].

### 2.8. Thermodynamic Properties

**Figure 11:**
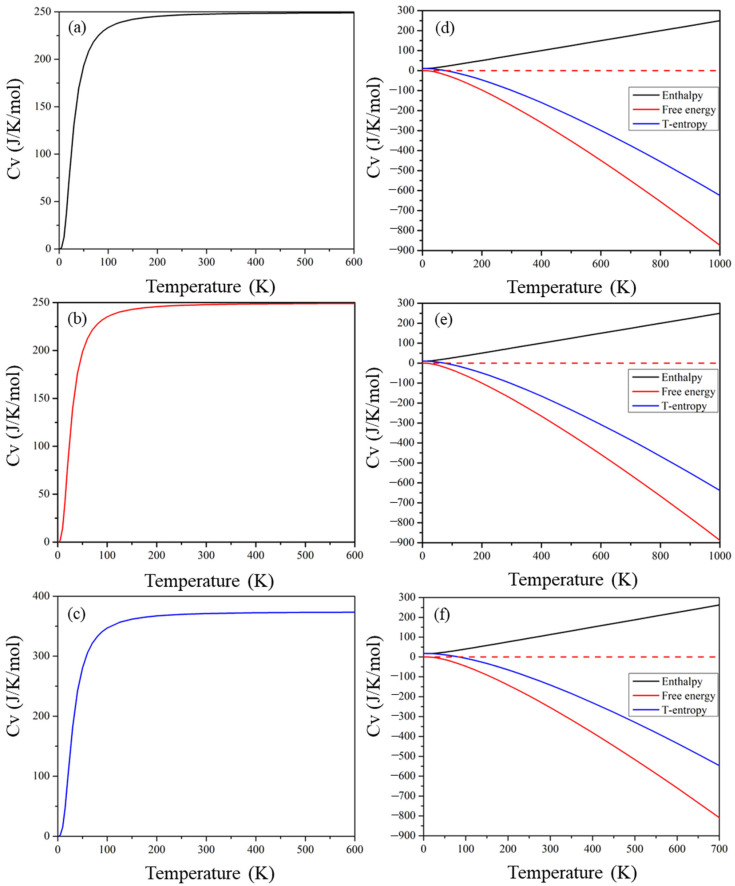
Temperature dependence of heat capacity and enthalpy, temperature entropy, and free energy of (**a**,**d**) Cs_2_Pb_2_I_3_Br_3_, (**b**,**e**) Cs_2_Pb_2_I_5_Br, and (**c**,**f**) Cs_3_Pb_3_IBr_8_ perovskites.

The calculated entropy (S), free energy (F), enthalpy (H), and heat capacity against temperature are presented in Figure 11. Entropy refers to the level of disorder of a material, and the middle lines in Figure 11d–f demonstrate the product of entropy and temperature for these materials. The thermal motion of molecules increases as the temperature rises, causing a high degree of disorder and entropy. The top solid lines in Figure 11d–f demonstrate materials’ enthalpy, which increases with increasing temperature as all three quantities (internal energy (U), volume (V), and pressure (P)) increase. The bottom three curves in Figure 11 show the free energy of the compound, expressed as F = U − TS. Both U and TS increase with increasing temperature; the increase in TS is greater, resulting in an increase in the value of TS − H. However, the value of H-TS decreases as the temperature increases and, hence, F = U − TS decreases with increasing temperature. The heat capacity of a material is composed of contributions from lattice vibration and electron motion, and Figure 11 depicts the variation in heat capacity with temperature. The melting point of these perovskite compounds increases rapidly below 200 K. At higher temperatures, the melting point increases gradually until it reaches the Dulong–Petit limit.

## 3. Computational Methods

### 3.1. Cluster Expansion

To determine the structural properties and stability of CsPbI_3−x_Br_x_ systems, the total energy for all the potential configurations must be calculated. Cluster expansion accounts for the energies of various atomic configurations by generating an Ising-like Hamiltonian and was used to calculate the configurational total energy. The cluster-expansion method was implemented within the UNCLE code [30]. The UNCLE code progressively increases the number of clusters considered in the cluster expansion until an ideal degree of precision is attained. This method was employed to determine the ground-state structure and the phase diagram of CsPbI_3−x_Br_x_ systems. The cross-validation score is provided as an indicator to assess the accuracy of the cluster-expansion fit.

### 3.2. Monte Carlo

The UNCLE code [30] was employed to perform the Monte Carlo simulations for CsPb(I/Br)_3_ systems. To achieve the desired precision in the average concentration of the CsPb(I/Br)_3_ compounds, the averaging times were set to 0.1%. The Monte Carlo moves involved exchanging the positions of I and Br atoms. The phase diagrams were calculated using the canonical ensemble (NVT) with specific parameters such as system volume (V), number of particles (N), and temperature (T), as implemented in the UNCLE code. The simulations used a supercell of 25 × 25 × 25 atoms. The temperature range explored in the simulations was set at 100 K to 3200 K.

### 3.3. Geometry Optimization

The first principle DFT was employed to optimize and calculate the properties of predicted structures implemented within VASP [31,32,33,34]. GGA was used as an exchange-correlation functionals [35]. The kinetic energy cut-offs were 520, 550, and 500 eV with the k-points of 4 × 4 × 5, 5 × 4 × 5 and 5 × 5 × 6 for the cluster-expansion predicted structures of Cs_2_Pb_2_I_3_Br_3_, Cs_2_Pb_2_I_5_Br, and Cs_3_Pb_3_IBr_8_, respectively. The convergence threshold for the total energy was set at 10^−6^ eV/atom. The compounds were considered fully optimized when forces were smaller than 10^−4^ eV/A.

## 4. Conclusions

The combined methods, including cluster expansion, Monte Carlo, and density functional theory, were employed to study the ground state search and phase stability of (CsPb)_x_I_y_Br_z_ (where x = 2 or 3 and y and z = 1 to 8) materials. To conduct Monte Carlo calculations, a set of effective cluster interactions, capable of providing an accurate formation energy for ordered CsPbI_1−x_Br_x_ configurations, was initially established within the cluster-expansion framework. There were 42 new ordered phases that were generated from a binary phase diagram. These newly generated phases demonstrated a miscibility gap because of thermodynamically unstable and phase separation. To further study the thermodynamic properties and phase separation at different temperatures for CsPbI_1−x_Br_x_ at 50% concentrations, the Monte Carlo method was used. The electronic properties showed that Cs_2_Pb_2_I_3_Br_3_, Cs_2_Pb_2_I_5_Br, and Cs_3_Pb_3_IBr_8_ are narrow band gap semiconductors with energy band gap values between 1.60, 1.59, and 1.90 eV, respectively. The optical properties demonstrated a strong ability of photon absorption because of their narrow band gaps. In all these predicted perovskite materials, specific heat capacity at low temperature obeys Debye’s law and at high temperature approaches the Dulong–Petit limit. The comparative analysis of predicted structures with CsPbI_3_ and CsPbBr_3_ showed that the opto-electronic properties of the perovskite compounds are enhanced with the mixed halides and can be used in solar cell applications.

## Figures and Tables

**Table 1:** The development of an iterative procedure that lists stable structures constructed of numerous pure phases.

No. of Iterations	No. of Structures	No. of New Structures	CSV Score (meV/pos)
0	0	2	-
0	0	12	-
1	12	22	0.026
2	22	10	0.461
3	32	10	0.603
4	42	10	0.652

**Table 2:** The calculated lattice parameters, angles, and bond lengths of (a) Cs_2_Pb_2_I_3_Br_3_, (b) Cs_3_Pb_3_IBr_8_, and (c) Cs_2_Pb_2_I_5_Br.

	Cs_2_Pb_2_I_3_Br_3_	Cs_2_Pb_2_I_5_Br	Cs_3_Pb_3_IBr_8_
Lattice constant (Å)	a = 11.973 b = 12.412 c = 6.403	a = b = 9.044 c = 6.206	a = 5.987 b = 17.967 c = 5.152
Volume (Å)	V = 951.421	V = 507.570	V = 661.663
Angle (°)	α= β = γ = 90.00	α= β = γ = 90.00	α= β = γ = 90.00
Bond length (Å)	Pb − I = 3.207 Pb − Br = 2.999	Pb − I = 3.201 Pb − Br = 3.001	Pb − I = 3.203 Pb − Br = 3.002
Band gap (eV)	1.692	1.520	1.901

**Table 3:** The calculated elastic constants C_ij_ of (Cs_2_Pb_2_I_3_Br_3_, Cs_2_Pb_2_I_5_Br, and Cs_3_Pb_3_IBr_8_) structures.

Elastic Constants	Cs_2_Pb_2_I_3_Br_3_	Cs_2_Pb_2_I_5_Br	Cs_3_Pb_3_IBr_8_
C_11_	41.921	23.290	39.602
C_12_	6.393	16.494	6.111
C_13_	5.960	5.163	5.242
C_22_	41.782	-	37.723
C_23_	5.961	-	4.843
C_33_	40.373	35.890	36.921
C_44_	3.972	-	3.451
C_55_	4.020	4.221	3.794
C_66_	4.431	3.751	4.673

**Table 4:** The calculated bulk, shear, and Young’s modulus (Cs_2_Pb_2_I_3_Br_3_, Cs_2_Pb_2_I_5_Br, and Cs_3_Pb_3_IBr_8_) structures.

Elastic Constants	Cs_2_Pb_2_I_3_Br_3_	Cs_2_Pb_2_I_5_Br	Cs_3_Pb_3_IBr_8_
Bulk	17.850	16.161	15.231
Shear	7.742	7.233	6.770
Young	20.171	18.771	17.590
Poisson	0.311	0.305	0.306
Pugh	2.306	2.235	2.248

## Data Availability

All data generated or analyzed during this study are included in this published article.
